# Analysis and experimental validation of fatty acid metabolism-related genes prostacyclin synthase (PTGIS) in endometrial cancer

**DOI:** 10.18632/aging.205080

**Published:** 2023-10-04

**Authors:** Bo Wang, Shuwen Ge, Zihao Wang, Wantong Wang, Yuting Wang, Hongrui Leng, Xiaoxin Ma

**Affiliations:** 1 Department of Obstetrics and Gynecology, Shengjing Hospital of China Medical University, Shenyang 110000, Liaoning, People’s Republic of China

**Keywords:** endometrial carcinoma (EC), fatty acid metabolism-related genes (FAMGs), prognostic model, tumor microenvironment (TME), prostacyclin synthase (PTGIS)

## Abstract

The deregulation of fatty acid metabolism plays a pivotal role in cancer. Our objective is to construct a prognostic model for patients with endometrial carcinoma (EC) based on genes related to fatty acid metabolism-related genes (FAMGs). RNA sequencing and clinical data for EC were obtained from The Cancer Genome Atlas (TCGA). Lasso-Penalized Cox regression was employed to derive the risk formula for the model, the score = e^sum(corresponding coefficient × each gene’s expression)^. Gene set enrichment analysis (GSEA) was utilized to examine the enrichment of KEGG and GO pathways within this model. Correlation analysis of immune function was conducted using Single-sample GSEA (ssGSEA). The “ESTIMATE” package in R was utilized to evaluate the tumor microenvironment. The support vector machine recursive feature elimination (SVM-RFE) and randomforest maps were employed to identify key genes. The effects of PTGIS on the malignant biological behavior of EC were assessed through CCK-8 assay, transwell invasion assay, cell cycle analysis, apoptosis assay, and tumor xenografts in nude mice. A novel prognostic signature comprising 10 FAMGs (INMT, ACACB, ACOT4, ACOXL, CYP4F3, FAAH, GPX1, HPGDS, PON3, PTGIS) was developed. This risk score serves as an independent prognostic marker validated for EC. According to ssGSEA analysis, the low- and high-risk groups exhibited distinct immune enrichments. The key gene PTGIS was screened by SVM-RFE and randomforest method. Furthermore, we validated the expression of PTGIS through qRT-PCR. *In vitro* and *in vivo* experiments also confirmed the effect of PTGIS on the malignant biological behavior of EC.

## INTRODUCTION

Endometrial cancer (EC) exhibits the highest prevalence among gynecological malignancies in the USA, Europe, and specific developed regions. While a significant number of EC patients can be diagnosed at an early stage, thereby enhancing their chances of survival through effective treatment, the prognosis for patients with advanced EC is often unfavorable [1, 2]. Hence, the identification of a novel molecular biomarker that enables precise diagnosis, treatment, and prognosis for EC is of utmost importance.

Metabolic dysregulation is a prominent characteristic of tumors and exerts a crucial influence on tumor initiation and progression [3–5]. Fatty acid metabolism, functioning as a pivotal intracellular process, facilitates the conversion of nutrients into indispensable metabolic intermediates, which contribute to membrane biosynthesis, energy reservoir, and the synthesis of pivotal signaling molecules [6]. Alterations in fatty acid metabolism represent a significant metabolic phenotype observed in tumor cells. Impeding the lipid supply to tumor cells profoundly impacts their bioenergetic status, membrane biogenesis, and intracellular signaling cascades [7–9]. Furthermore, this perturbation also exerts an impact on diverse aspects such as tumor cell migration, initiation of angiogenic processes, establishment of metabolic symbiosis, evasion of immune surveillance, and development of resistance to therapeutic agents within tumor microenvironments. This phenomenon assumes a significant role not only in liver and breast cancer but also in various other malignancies [10, 11]. Nevertheless, the prognostic significance of genes associated with fatty acid metabolism in EC, as well as their association with immunotherapy and chemotherapy treatment, remains largely elusive.

In our investigation, we conducted a comprehensive analysis of prognostic genes associated with fatty acid metabolism and differentially expressed groups related to fatty acid metabolism. Subsequently, we employed LASSO regression to build a prognostic model. The nomogram incorporated both the risk scores generated by the model and clinical data of EC patients. The prognostic model demonstrated its status as an independent prognostic factor for EC, exhibiting strong predictive capability and outperforming existing models in terms of predictive efficacy. By utilizing support vector machine recursive feature elimination (SVM-RFE) and randomforest maps, we successfully identified prostacyclin synthase (PTGIS) as a key gene within the model. To further validate the role of PTGIS in EC, we conducted *in vivo* and *in vitro* experiments, providing novel insights into prognosis and precision medicine in EC.

## RESULTS

**Figure 1:**
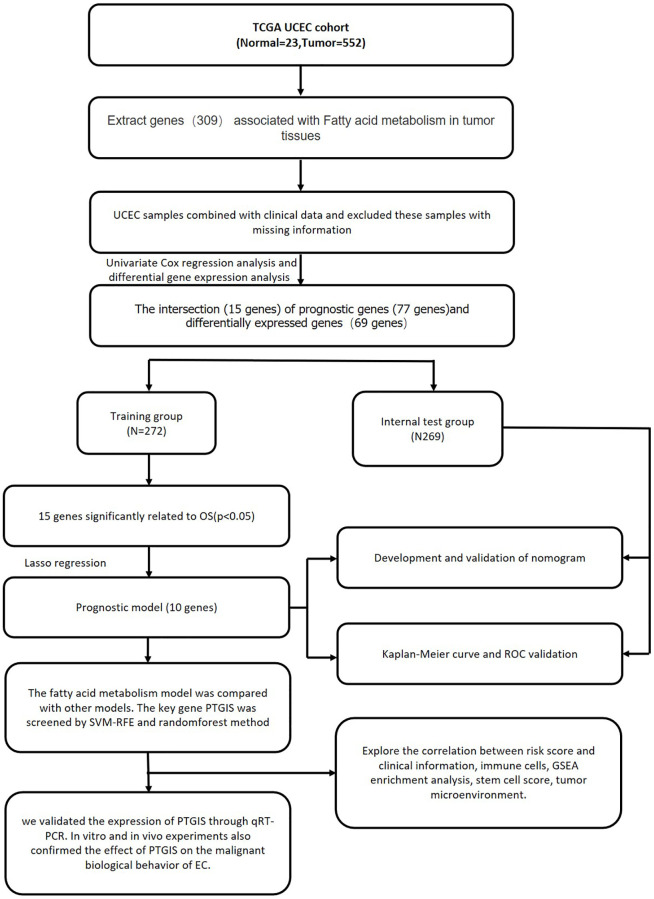
Comprehensive prognostic value analysis framework of fatty acid metabolism-related genes (FAMGs) in uterine corpus endometrial carcinoma (UCEC) patients based on TCGA database.

Our study initially developed an advanced prognostic model for EC utilizing genes associated with fatty acid metabolism, surpassing the performance of existing models. Subsequently, we focused on investigating the role of the key gene PTGIS within this model using SVM and random forest methods. Our subsequent experiments provided substantial evidence supporting the impact of PTGIS on the malignant biological behavior of EC. Consequently, PTGIS emerges as a promising candidate worthy of consideration as a prospective therapeutic target in EC (Figure 1).

### Construction of a prognostic model

**Figure 2:**
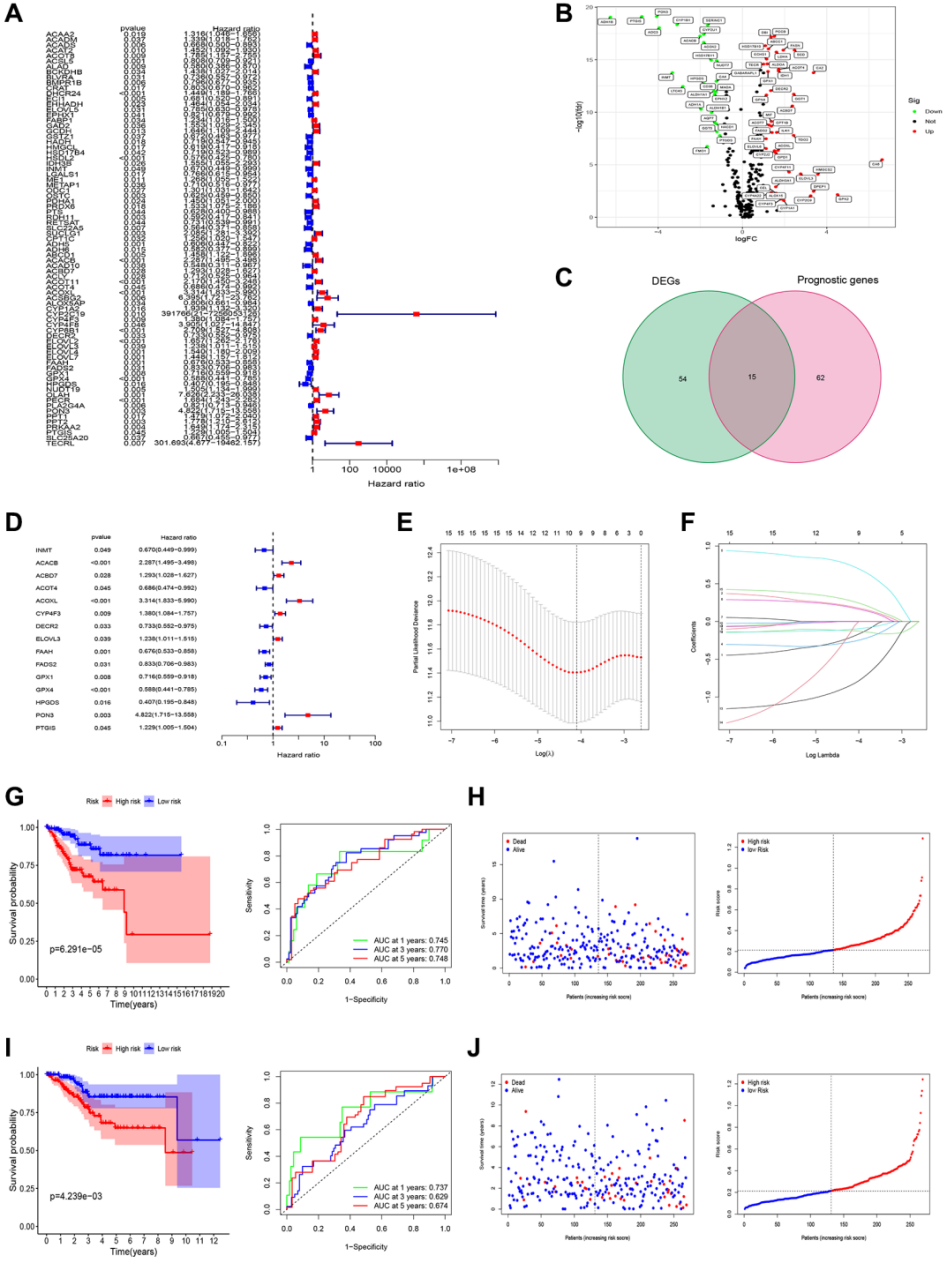
**Screening of prognosis FAMGs and construction of prognosis model.** (**A**) Univariate Cox regression analysis to identify the candidate prognosis-related hub LMGs in UCEC. (**B**) volcano plot of Differentially expressed LMGs (DEFAMGs): upregulated DELMGs are indicated by red dots, and downregulated DELMGs are indicated by green dots. (**C**) The DEIRGs were intersected with the prognosis-related LMGs. (**D**) A univariate analysis of the intersection genes was obtained. (**E**) Partial likelihood deviation was plotted relative to the logarithm of lambda in 10-fold cross-validation. (**F**) The trajectory graph of each variable. (**G**) Survival curves and ROC curves of high and low risk groups in the training group. (**H**) The risk score value of each sample, the survival status ranked from low to high-risk scores in the training group. (**I**) Survival curves and ROC curves of high and low risk groups in the test group. (**J**) The risk score value of each sample, the survival status ranked from low to high-risk scores in the test group.

**Figure 3:**
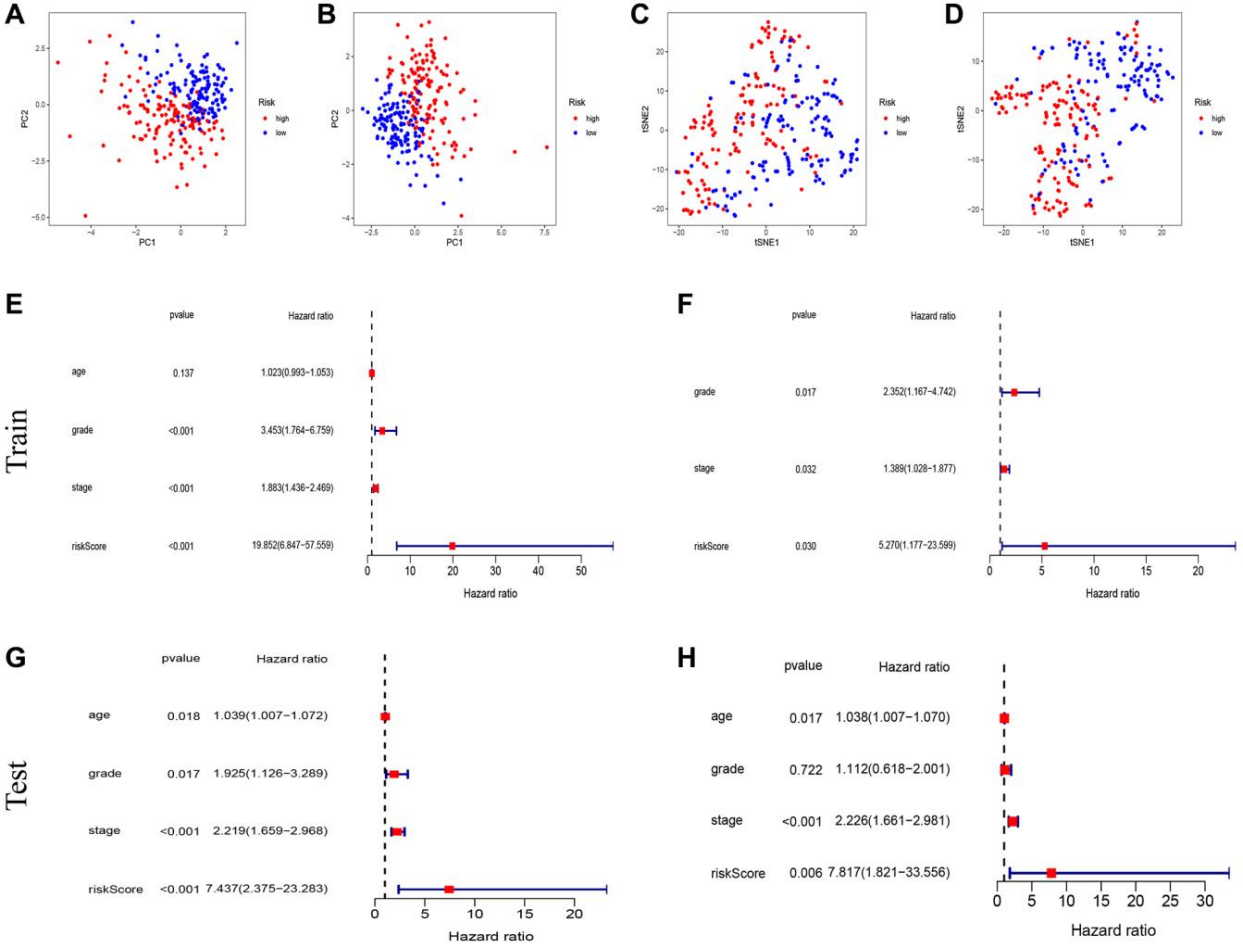
**Test of risk prediction model for UCEC patients.** PCA plot for (**A**) training sets and (**B**) test sets. T-SNE analysis for (**C**) training sets and (**D**) test sets. (**E**, **F**) Univariate and multivariate analysis were performed to assess the clinicopathological prognostic value of the prediction model in the training group. (**G**, **H**) Univariate and multivariate analysis were performed to assess the clinicopathological prognostic value of the prediction model in the test group.

In the univariate Cox regression analysis, it was observed that 77 out of 309 fatty acid metabolism-related genes (FAMGs) exhibited associations with overall survival (OS) (Figure 2A). Vol map was used to identify 69 differentially expressed FAMGs (DEFAMGs) (Figure 2B). The intersection of DEFAMGs and Prognostic associated FAMGs resulted in 15 common genes, as depicted in Figure 2C, 2D. To further refine the selection, Lasso-Cox regression analysis was employed, leading to the identification of these 15 common genes (Figure 2E, 2F). Risk score = e^((−0.189 × Exp (INMT)) + (0.188 × Exp (ACACB)) + (−0.260 × Exp (ACOT4)) + (0.722 × Exp (ACOXL)) + (0.175 × Exp (CYP4F3)) + (−0.124 × Exp (FAAH)) + (−0.101 × Exp (GPX1)) + (−0.818 × Exp (HPGDS)) + (−0.054 × Exp (PON3)) + (0.260 × Exp (PTGIS)))^. Kaplan-Meier (KM) analysis demonstrated that patients with high-risk scores exhibited significantly worse outcomes in the training group (Figure 2G). The area under the receiver operating characteristic (ROC) curves (AUCs) for 1-, 3-, and 5-year OS was calculated as 0.745, 0.770, and 0.748, respectively, in the training group (Figure 2G). Figure 2H depicted the distribution of risk scores and survival status among patients in the training group. We did the same analysis in the test group. The same analysis was conducted in the test group, where the risk score also demonstrated robust predictive ability (Figure 2I, 2J). Furthermore, Principal Component Analysis (PCA) and t-SNE analysis revealed that the model effectively differentiated between high and low-risk groups in both the training set (Figure 3A, 3B) and the test set (Figure 3C, 3D). Subsequent validation through univariate and multivariate analyses consistently corroborated the autonomous independent prognostic significance of the established risk score, substantiated across in both the training set (Figure 3E, 3F) and the test set (Figure 3G, 3H).

### Establishment of the nomogram

**Figure 4:**
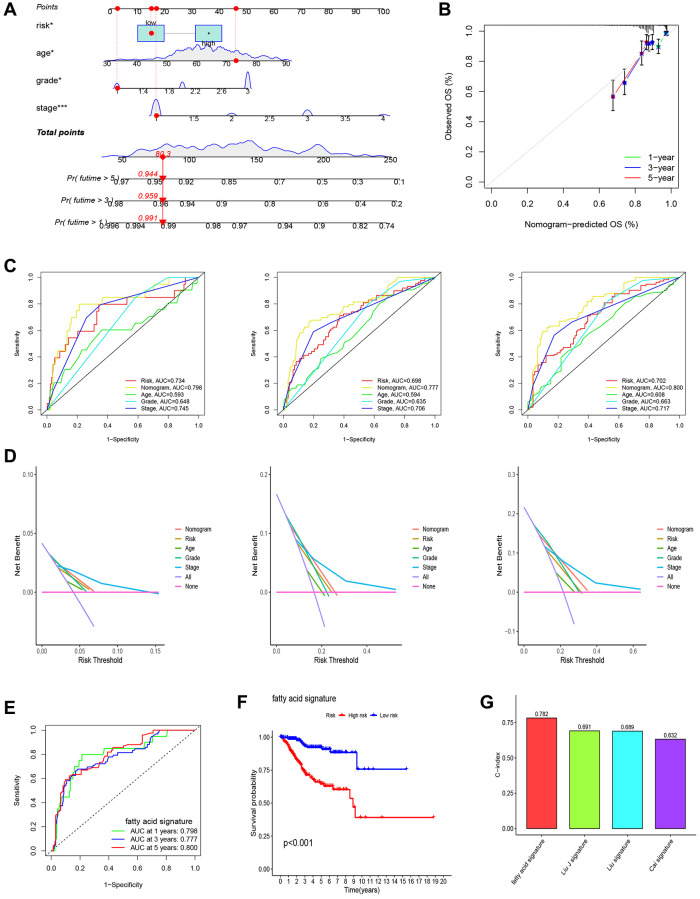
**Establishment of nomogram and comparison with existing models.** (**A**) Nomogram for predicting the 1-, 3-, and 5-year OS of UCEC patients. (**B**) Calibration curves for the prediction of 1-, 3- or 5-year overall survival of UCEC patients. (**C**) ROC curves for predicting the 1-, 3-, and 5-year OS of UCEC patients. (**D**) Decision Curve Analysis (DCA) curves for predicting the 1-, 3-, and 5-year OS of UCEC patients. (**E**, **F**) Survival curves and ROC curves of high and low risk groups in the model constructed by us. (**G**) C-index comparison of inflammatory models with other models.

To enhance the predictive capability of our model, we further developed a nomogram, as depicted in Figure 4A. The calibration curves demonstrated the accuracy and validity of the nomogram (Figure 4B). The AUCs for 1-, 3-, and 5-year OS using the nomogram were calculated as 0.798, 0.777, and 0.800, respectively (Figure 4C). Decision curve analysis (DCA) results show that nomogram has good prediction efficiency (Figure 4D). KM analysis and ROC curves also illustrated the improved predictive power of the nomogram (Figure 4E, 4F). Additionally, the C-index results indicated that our constructed model outperformed the three other models developed by Cai, Liu, and Liu J [12–14] (Figure 4G).

### Association analysis of risk score

**Figure 5:**
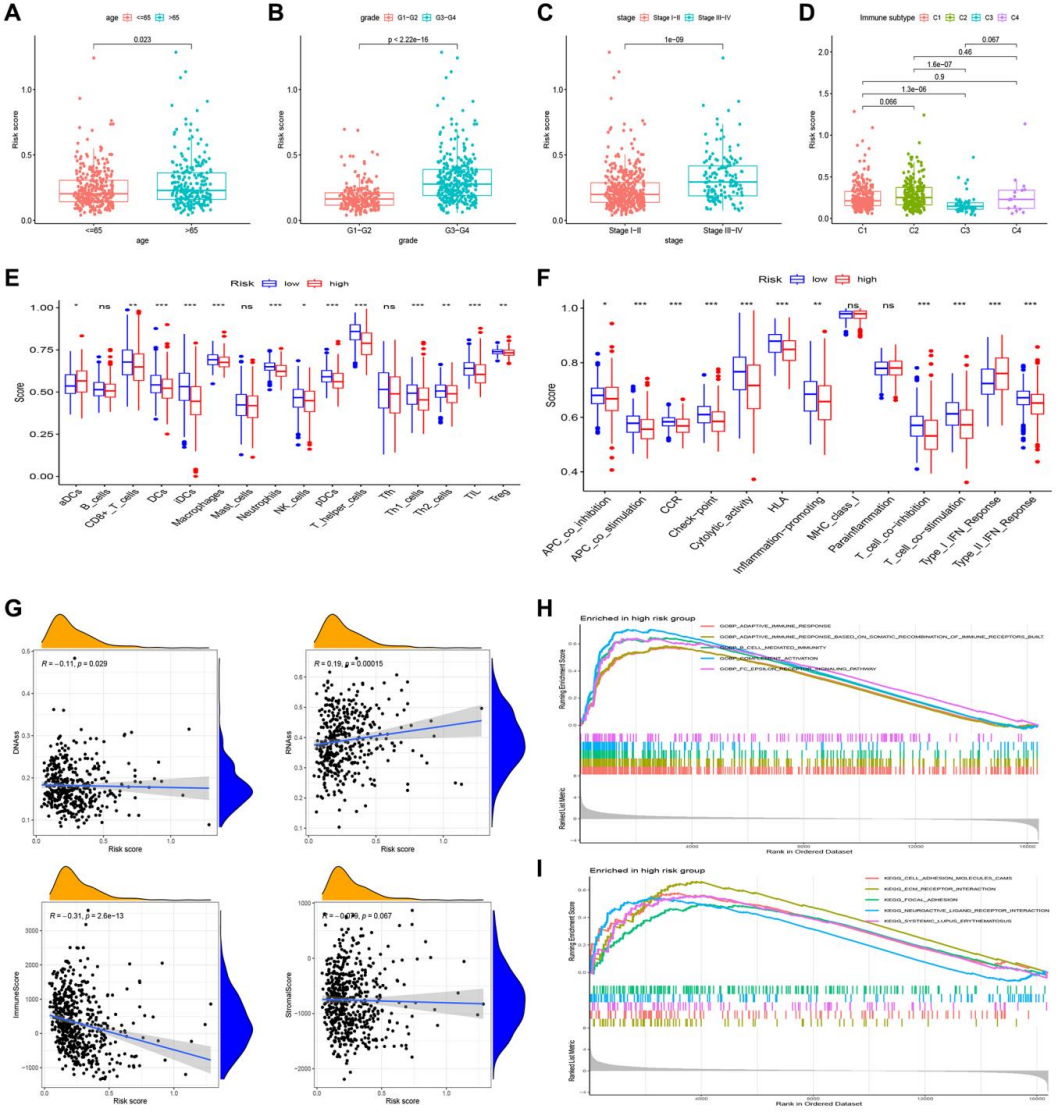
**Gene set enrichment analysis (GSEA) of biological functions and the association between risk score and tumor microenvironment.** The risk score in different groups divided by age (**A**), grade (**B**), stage (**C**) and immune subtype (**D**). Comparison of the risk score in different immune infiltration. (**E**, **F**) The relationship between risk score and the scores of 16 immune cells and 13 immune-related functions were showed in boxplots. (**G**) The relationship between risk score and DNAss, RNAss, Stromal Score and Immune Score. (**H**, **I**) GSEA showed eleven pathways enriched in the high-risk group. *P* values were showed as: Abbreviation: ns: not significant; ^*^P < 0.05; ^**^P < 0.01; ^***^P < 0.001.

In our analysis, we conducted a further investigation into the correlation between risk scores and various clinicopathological parameters as well as immune subtyping. The correlation analysis revealed that the higher age group (>65 years), higher differentiation group (tumor grade 3–4), and higher clinical stage (tumor stage III–IV) exhibited higher risk scores (Figure 5A–5C). Additionally, we observed that the immune subtype associated with a poor prognosis (C2) displayed a higher risk score compared to the immune subtype linked to a favorable prognosis (Figure 5D).

Single-sample Gene Set Enrichment Analysis (ssGSEA) revealed that most of the immune cells and immune function were suppressed in the high-risk group, and fractions of CD8^+^T cells, DCs, iDCs, Macrophages, Neutrophils, pDCs, T helper cells, Th1 cells, and TIL were significantly decreased (Figure 5E). Moreover, APC, CCR, check-point, and HLA were lower in high-risk group. (Figure 5F).

In evaluating cancer stem cells (CSCs), DNA stemness score (DNAss) and RNA stemness score (RNAss) were utilized [15]. Correlation analysis revealed a positive correlation between the risk score and RNAss, indicating that higher risk scores corresponded to stronger stem cell characteristics in tumor cells (Figure 5G). To assess the tumor microenvironment (TME), immune and stromal scores were employed. Correlation analysis demonstrated a negative correlation between the risk score and immune score (Figure 5G).

In our study, we performed pathway enrichment analysis specifically for the high-risk group. The GSEA enrichment analyses revealed significant enrichments in various pathways. In the high-risk group, pathways such as adaptive immune response, mediated immunity, complement activation, B cell-mediated immunity, cell adhesion molecules, ECM receptor interaction, focal adhesion, neuroactive ligand-receptor interaction, and systemic lupus erythematosus exhibited significant enrichments (Figure 5H, 5I).

### Screening of key genes in the model

**Figure 6:**
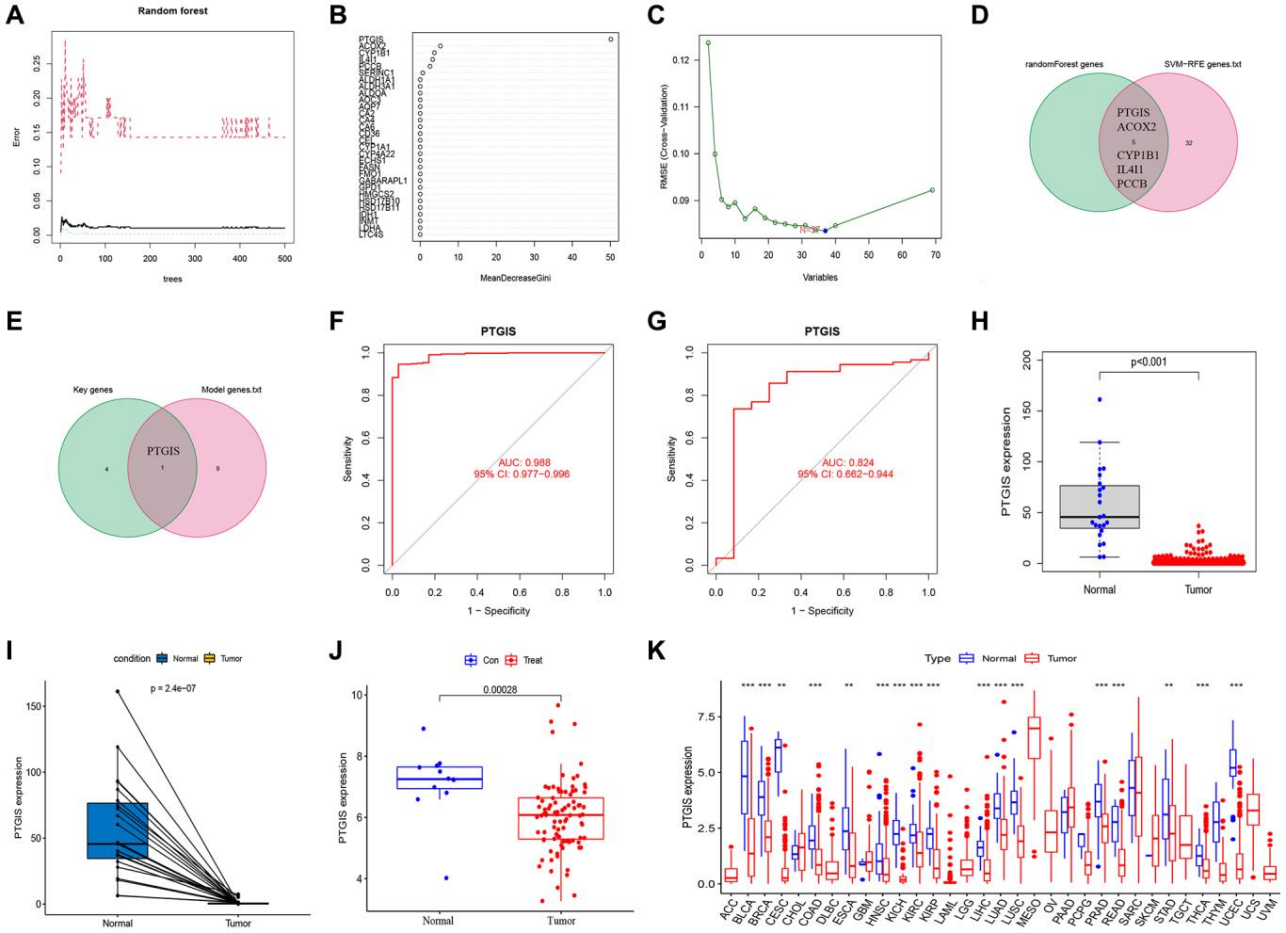
**Feature gene selection.** (**A**, **B**) RandomForest error rate versus the number of classification trees. (**C**) Biomarker signature gene expression validation by support vector machine recursive feature elimination (SVM–RFE) algorithm selection. (**D**) The intersection genes of SVM-RFE and RandomForest were screened by Venn diagram. (**E**) The genes included in our model were intersected with key characteristic genes to obtain PTGIS. (**F**) The ROC curve of PTGIS predicted the incidence of UCEC in TCGA database. (**G**) The ROC curve of PTGIS predicted the incidence of EC in GSE17025. (**H**) Box plots showed the expression of PTGIS in normal and UCEC tissues from TCGA. (**I**) The transcription levels of PTGIS in UCEC compared with the paired normal endometrial tissue was showed based on TCGA datasets. (**J**) Box plots showed the expression of PTGIS in normal and EC tissues from GSE17025. (**K**) Expression of PTGIS in pan-cancer.

We utilized random forest analysis to screen the 309 FAMGs and identified genes relevant to EC (Figure 6A, 6B). The SVM-RFE algorithm was employed to select the expression of feature genes from the 309 FAMGs (Figure 6C). By intersecting the results of random forest analysis with those of SVM-RFE, we identified 5 key characteristic genes (PTGIS, ACOX2, CYP1B1, IL4I1, PCCB) (Figure 6D). From these 5 key feature genes, we further intersected them with the genes involved in model construction and identified PTGIS as the key gene within the model (Figure 6E). The AUC of PTGIS as a diagnostic gene was determined as 0.988 in the TCGA-UCEC database (Figure 6F). To validate the diagnostic capability of PTGIS for EC, we obtained the external dataset GSE17025 and found that the AUC of PTGIS was 0.824 in this dataset (Figure 6G). Additionally, we observed down-regulated expression of PTGIS in both the TCGA-UCEC dataset (Figure 6H, 6I), and the GSE17025 dataset, further confirming its association with EC (Figure 6J). Furthermore, in pan-cancer analysis, it was consistently observed that PTGIS was down-regulated in most tumors (Figure 6K).

### Experimental validation of PTGIS

**Figure 7:**
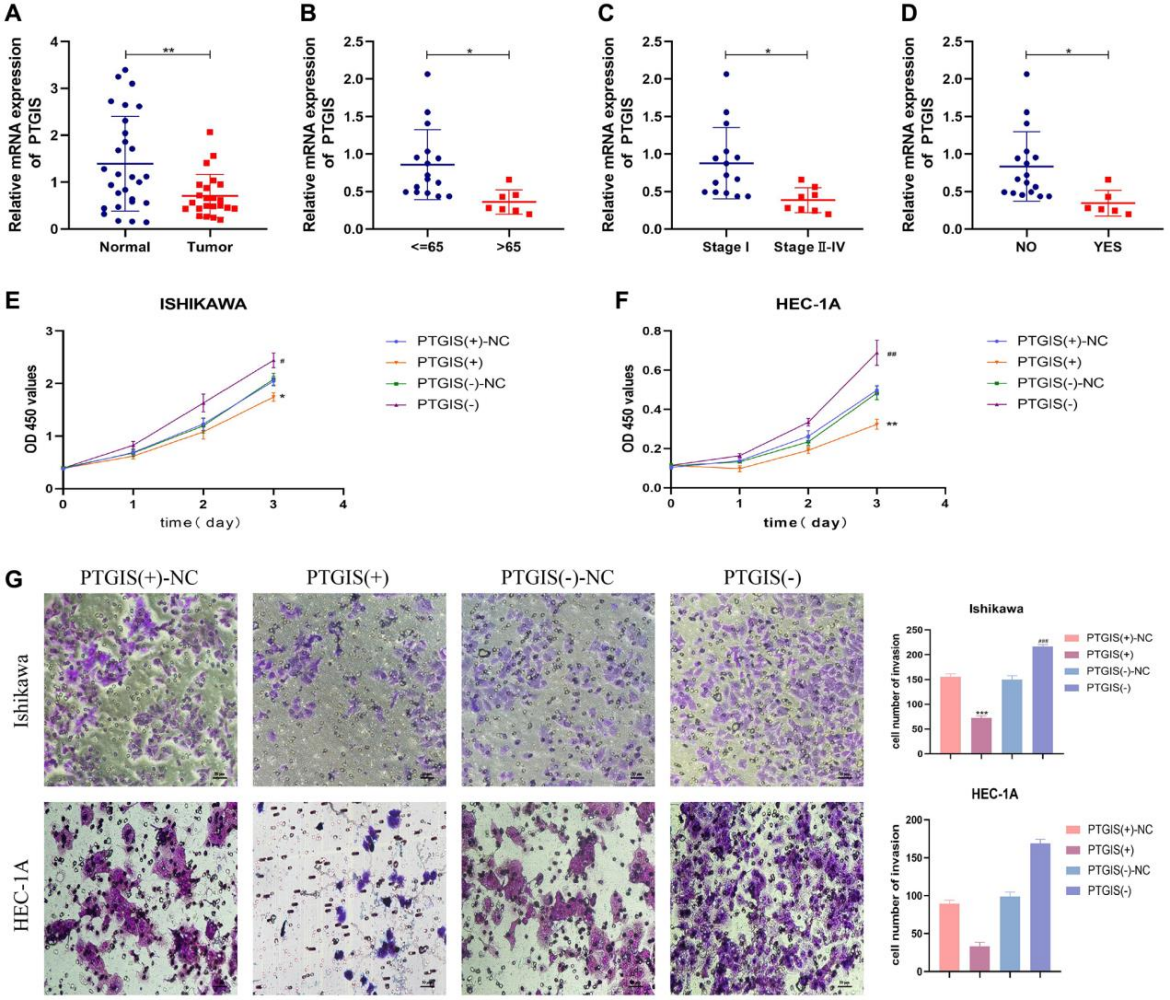
**PTGIS regulates the biological behavior of EC cell lines.** (**A**) he results of qRT-PCR showed the expression of PTGIS in normal endometrial tissue (*n* = 28) and human endometrial carcinoma tissue (*n* = 23). The expression of PTGIS in different groups divided by age (**B**), stage (**C**) and LN-metastasis (**D**). (**E**, **F**) CCK-8 assay was used to evaluate the proliferation effect of PTGIS. (**G**) Effect of LAMP3 on invasion assessed using the Transwell assay.

The qRT-PCR results demonstrated down-regulation of PTGIS expression in EC (Figure 7A). Additionally, we conducted an analysis to examine the relationship between PTGIS expression and clinicopathological parameters. The findings indicated that PTGIS expression was decreased in the >65 age group, tumor stage III–IV group, and lymph node (LN) metastasis group (Figure 7B–7D).

**Figure 8:**
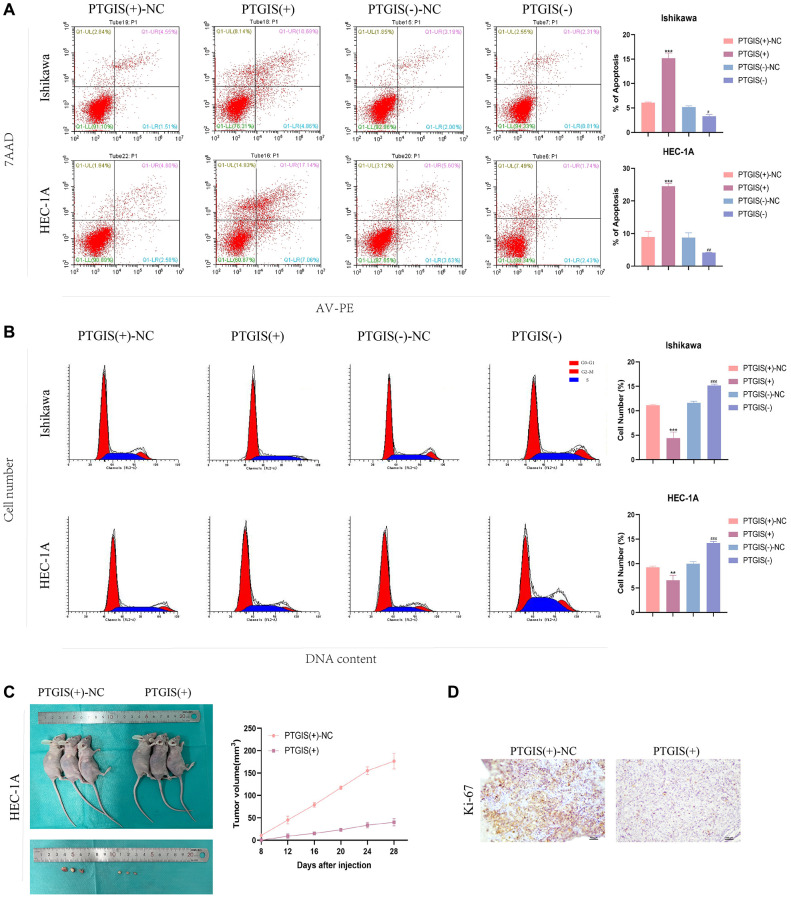
**PTGIS regulates the biological behavior of EC cell lines and *in vivo* study of tumor xenografts.** (**A**) Cell apoptosis assay was used to determine the effect of PTGIS on the apoptosis of Ishikawa and HEC-1A cell lines. (**B**) Cell cycle analysis was used to detect the effect of PTGIS on the cell cycle of Ishikawa and HEC-1A cell lines. (**C**) The nude mice carrying tumors from the respective groups are shown. The sample tumors from the respective groups are shown (*n* = 3, each group). (**D**) Expression levels of Ki-67.

To evaluate the impact of PTGIS on cellular functions, we performed the CCK-8 assay to assess cell proliferation. The results demonstrated that overexpression of PTGIS inhibited cell proliferation, whereas reducing PTGIS expression enhanced cell proliferation in ISHIKAWA and HEC-1A cells (Figure 7E, 7F). Furthermore, overexpression of PTGIS decreased cell invasion ability, while decreased PTGIS expression promoted cell invasion (Figure 7G). Apoptosis experiments revealed that overexpression of PTGIS promoted apoptosis, whereas knockdown of PTGIS inhibited apoptosis (Figure 8A). Cell cycle analysis demonstrated that compared to the PTGIS (+)-NC group, the PTGIS (+) group exhibited a decrease in the number of cells in the G2-M phase of the cell cycle. Conversely, compared to the PTGIS (−)-NC group, the PTGIS (−)-NC group displayed an increased proportion of cells in the G2-M phase in both ISHIKAWA and HEC-1A cells (Figure 8B). The outcomes of the tumor formation experiment conducted in nude mice distinctly demonstrated that the overexpression of PTGIS yielded a notable suppression in tumor growth, as evidenced by a significant contrast between the PTGIS (+) group and the PTGIS (+)-NC group (Figure 8C). Immunohistochemical results showed that the KI-67 staining score of PTGIS (+) group was lower than that of PTGIS (+)-NC, further indicating that overexpression of PTGIS inhibited the growth of EC (Figure 8D).

## DISCUSSION

In developed countries, the incidence of EC has been steadily increasing, making it the most prevalent gynecological tumor in women [16–18]. Fortunately, due to the detectability of postmenopausal bleeding, most cases of endometrial cancer are diagnosed at an early stage, allowing for prompt and effective surgical treatment. This has resulted in a 5-year survival rate of nearly 95% for EC patients. However, in advanced stages of EC, where metastasis and invasion occur, the five-year survival rate drops to 16–45% [2]. The incidence of EC has been on the rise, with an average annual increase of 1.9%. This increase can primarily be attributed to the growing prevalence of obesity [19, 20]. Therefore, it is crucial to identify new therapeutic targets and prognostic markers for EC to improve patient outcomes.

Metabolic dysfunction is a significant characteristic of tumors, and the disruption of fatty acid metabolism plays a critical role in this process. Recent studies have highlighted the potential of targeting fatty acid metabolic pathways for drug therapy and immunotherapy in various types of tumors [21–23]. Fatty acid metabolism is involved in energy synthesis, production of signaling molecules, and regulation of the tumor microenvironment, thereby influencing tumor progression [24]. Although the significance of fatty acid metabolism has been extensively investigated in the context of breast and cervical cancer, there exists a noticeable dearth of research concerning the underlying mechanisms governing fatty acid metabolism in the initiation and advancement of EC [25, 26]. Consequently, the primary aim of this study was to establish a prognostic model for EC based on genes associated with FAMGs and identify significant therapeutic targets for EC.

In the analysis using TCGA-UCEC data, we performed univariate analysis on the 309 FAMGs and identified 77 FAMGs that were relevant to prognosis. Further differential expression analysis resulted in 69 differentially expressed FAMGs. By intersecting these differentially expressed genes with the prognostic genes, we obtained 15 common genes. Through Lasso-Cox regression analysis, we refined the selection to obtain a prognostic model comprising 10 genes: INMT, ACACB, ACOT4, ACOXL, CYP4F3, FAAH, GPX1, HPGDS, PON3, and PTGIS. The high-risk group identified by the model exhibited a worse prognosis, and the risk score derived from the model served as an independent prognostic factor. Nomograms were constructed to provide improved predictions of patient outcomes. The comparative analysis demonstrated that our model outperformed existing models. Correlation analysis between the risk score and clinicopathological parameters revealed a positive correlation between the risk score and clinical risk factors. Higher risk scores were associated with higher clinical risks factors such as stage III–IV, grade 3–4, and age >65. Furthermore, analysis of immune subtypes showed higher risk scores in the C1, C2, and C4 subtypes, which are known to be associated with poorer prognosis [27]. These findings support the conclusion that the high-risk group indeed exhibited a worse prognosis.

Many studies have demonstrated the impact of abnormal fatty acid metabolism on the tumor microenvironment, thereby promoting tumor progression [28, 29]. In our study, we observed that the high-risk group exhibited suppression of immune cells (CD8^+^T cells, DCs, iDCs, Macrophages, Neutrophils, pDCs, T helper cells, Th1 cells, and TIL) and immune function (APC, CCR, check-point, and HLA) compared to the low-risk group. The levels of these immune cells and functions represent the immunity level of each sample. These results suggest that abnormal fatty acid metabolism in EC may further enhance tumor progression by inhibiting immune function and immune response. Previous studies have indicated that abnormal fatty acid metabolism may be associated with resistance to immunotherapy in liver cancer [30]. Additionally, FDX1, a gene related to fatty acid metabolism, has been shown to regulate the progression of clear-cell renal carcinoma by influencing the immune microenvironment of tumor cells [31]. Furthermore, there is evidence linking fatty acid metabolism to immune checkpoints in melanoma [32]. In summary, fatty acid metabolism plays a significant role in immune regulation [33].

The correlation analysis results between the tumor microenvironment and risk score indicated that the risk score exhibited a positive correlation with RNAss and a negative correlation with immune score. It is well-established that CSCs possess the capability to self-renew and differentiate, contributing to cancer recurrence, chemotherapy resistance, and tumor progression. Emerging studies have revealed an association between fatty acid metabolism and tumor stem cells [34]. Abnormal fatty acid metabolism is believed to provide an enhanced energy supply to sustain the maintenance of tumor stem cells [35].

To further refine the selection of key genes in our model, we employed the SVM-RFE and random forest methods for joint analysis. This approach led us to identify PTGIS as a key gene in the model. As a diagnostic gene for EC, PTGIS exhibited an impressive AUC of 0.988 in the TCGA-UCEC dataset. Furthermore, validation using the external dataset GSE17025 yielded an AUC of 0.824 for PTGIS as a diagnostic gene. Notably, PTGIS not only played a crucial role in the prognostic model but also demonstrated substantial potential as a standalone diagnostic gene. Expression analysis revealed a down-regulation of PTGIS in EC as well as in several other tumor types. This suggests that PTGIS may function as a tumor suppressor gene and holds significance in the context of EC. To further substantiate the importance of PTGIS in EC, we conducted *in vivo* and *in vitro* experiments. qRT-PCR experiments performed on pathological tissues from Shengjing Hospital confirmed the reduced expression of PTGIS in EC. Furthermore, the findings derived from both *in vitro* and *in vivo* experiments offered supplementary substantiation, affirming the involvement of PTGIS in impeding the advancement of cancer within the scope of EC.

PTGIS serves as an enzyme responsible for the synthesis of prostacyclin, a crucial mediator in vascular dilation and anticoagulant processes. Prior research has indicated that mutations within the PTGIS gene could potentially heighten the vulnerability to pulmonary hypertension [36]. Additionally, PTGIS methylation has been implicated in promoting liver fibrosis [37], and PTGIS has been found to protect hematopoietic stem cells in specific conditions [38]. More recently, studies have shed light on the involvement of PTGIS in various types of tumors. PTGIS has been implicated in the progression of stomach, ovarian, and lung cancers [39]. Furthermore, PTGIS is involved in regulating the malignant behavior of bladder cancer under hypoxic conditions [40]. In the context of endometriosis, PTGIS has been shown to regulate disease progression through its influence on CD16-NK cells, although no studies have been reported on PTGIS in the context of EC [41]. Our study not only provides a novel and effective predictive model for EC prognosis but also establishes a solid foundation for the investigation of fatty acid metabolism in EC. Importantly, our findings demonstrate that PTGIS can serve as an ideal diagnostic indicator and potentially acts as a tumor suppressor gene in EC. These discoveries position PTGIS as a promising candidate for future therapeutic interventions targeting EC.

This study does have certain limitations that need to be acknowledged. There was no external validation due to the lack of other datasets with UCEC clinical data. The applicability and effectiveness of the prognostic model in clinical practice require further validation, and future studies are planned to address these aspects. Nonetheless, this study represents the first successful construction of an EC prognostic model utilizing genes associated with fatty acid metabolism, allowing for accurate prediction of EC patient prognosis. Furthermore, we have identified PTGIS as a key gene within the model, highlighting its potential as a diagnostic, predictive, and therapeutic target for EC patients.

## CONCLUSION

The novel FAMGs-based model we have constructed demonstrates consistent and reliable predictive capability concerning patient prognosis within the context of EC. This model exhibits considerable potential as a prospective prognostic indicator for individuals diagnosed with EC, aiding in personalized cancer treatment and precision medicine. Furthermore, our identification of PTGIS as a key gene within the model highlights its potential as a target for EC diagnosis, prediction, and treatment. These findings carry important clinical implications, offering potential value in enhancing the management of prognosis for EC patients.

## MATERIALS AND METHODS

### Data acquisition

We collected clinical information and RNA sequencing datasets (FPKM) from the TCGA database for patients with uterine corpus EC (UCEC). The dataset comprised RNA sequencing data from 552 UCEC tissues and 35 normal tissues, along with clinical information for 541 patients. To identify the FAMGs, we selected genes from the Reactome fatty acid metabolism genes, KEGG fatty acid metabolism pathways, and Hallmark fatty acid metabolism genes databases (available at gsea-msigdb.org). After removing duplicates, we obtained a total of 309 unique FAMGs for further analysis [42].

### Identification of FAMGs and construction of model

In our study, a univariate Cox regression analysis was conducted encompassing the cohort of 309 FAMGs. For the identification of differentially expressed FAMGs, we employed the “Limma” package. As the TCGA dataset was the only source of prognostic information for EC patients available, our model could only be internally tested using this dataset. To conduct the analysis, we randomly divided the TCGA-UCEC patients into a training set (*n* = 272) and a test set (*n* = 269). Subsequently, we utilized LASSO-penalized Cox regression analysis in the training set to further refine our model [43]. The risk score = e^sum(each gene’s expression × corresponding coefficient)^. In the training group of UCEC patients, the prognosis data was stratified into high-risk and low-risk subgroups based on a predefined cutoff value. The validity of the established model was then verified using the test group. Additionally, we constructed a Nomogram that incorporated clinical information of EC patients and compared it with previously established models. To assess pathway enrichment, we performed GSEA analysis. Furthermore, the correlation between risk scores and patients’ immune function was evaluated using ssGSEA analysis [44]. For the purpose of screening key genes in the model, we utilized the R packages “randomForest”, “kernlab”, and “caret” for SVM and random forest map analysis.

### Tumor microenvironment analysis

We utilized the R package “ESTIMATE” to calculate immune and stromal scores for each sample. Subsequently, the “limma” package was employed to determine the correlation between risk scores and immune and stromal scores, respectively [44]. To assess the level of CSCs in each patient’s tumor, we downloaded epigenome and transcriptome data and performed correlation analyses to evaluate the relationship between risk scores and CSCs.

### Human tissue specimens

We collected a total of 28 normal endometrial tissues and 23 EC tissues from Shengjing Hospital of China Medical University, China, between 2019 and 2021.

Before being included in the study, all participants granted their informed consent, thus adhering to ethical practices. The clinicopathological attributes of all individuals were procured, and the histopathological characterization of all cases of endometrial carcinoma was definitively ascertained as endometrial adenocarcinoma. The pathological diagnosis of EC was established by two experienced pathologists following the guidelines set forth by the International Federation of Gynecology and Obstetrics (FIGO 2009). None of the patients received any form of hormone therapy, radiotherapy, chemotherapy, or other treatments prior to surgery.

### qRT-PCR

For RNA extraction, we utilized the TRIzol reagent (Vazyme, Nanjing, China) following the manufacturer’s instructions. Subsequently, cDNAs were synthesized using Prime Script RT-polymerase (Vazyme). The expression levels of the target genes were determined using SYBR Green Premix (Vazyme) along with specific PCR primers obtained from Sangon Biotech Co., Ltd., (Shanghai, China). The primer sequences can be found in Supplementary Table 1. Fold changes were calculated using the 2^(−ΔΔCT)^ method, which compares the relative expression levels between samples.

### Transfection of cells

PTGIS lentiviral overexpression were purchased from Hanbio Tech (Shanghai, China). SiRNA sequences targeting PTGIS were procured from GenePharma (Shanghai, China). Sequences of siRNA are listed in Supplementary Table 2. Lipofectamine 3000 (Invitrogen) was employed to effectuate the transfection of cells with the small interfering RNA (siRNA), as directed by the manufacturer’s stipulated guidelines.

### Cell culture

ISHIKAWA cells and HEC-1A cells were cultivated utilizing medium 1640 (Gibco, Carlsbad, CA, USA) and 5A (Gibco), respectively. A 10% concentration of fetal bovine serum (FBS) (Gibco) and 1% penicillin–streptomycin was supplemented to the cellular medium. Subsequently, all cells were nurtured in a humidified incubation chamber maintained at a temperature of 37°C with a 5% concentration of carbon dioxide (CO2).

### CCK-8 assay

Ishikawa and HEC-1A cells were seeded in 96-well plates, followed by the addition of CCK-8 reagent (10 μL) (Dojindo, Japan) to each well. Subsequently, the plates were incubated at a temperature of 37°C with a 5% concentration of carbon dioxide (CO2) for a duration of 3 hours. To determine the optical density (OD450) values, a microplate reader (Bio-Rad, Hercules, United States) was employed at four time points: 0 hours, 24 hours, 48 hours, and 72 hours post-treatment.

### Cell invasion assay

The TRANSWELL assay was employed to evaluate cell invasions. 8 μm Transwell chambers were pre-coated with a Matrigel solution prior to cell seeding. Subsequently, a 200 μl serum-free medium containing 2 × 10^4^ cells was introduced into the upper chamber, while a 500 μl solution of 10% FBS serum was added to the lower chamber. Following a 24-hour incubation period, the cells were fixed using 4% paraformaldehyde and stained with crystal violet, enabling visualization of the cells beneath the chamber through the Matrigel. Subsequently, images were captured using a fluorescent inverted microscope (NIKON, Japan) at a magnification of 200×, and subsequently subjected to analysis.

### Apoptosis assay

Following cell transfection, a total of 10^6^ cells from each experimental group were subjected to a PBS wash. Subsequently, the cells were incubated at room temperature, shielded from light, with PE Annexin V and 7AAD dye for a duration of 15 minutes. Flow cytometry analysis was performed using a Beckman DxFLEX instrument located in Suzhou, China to quantify the percentage of apoptotic cells within the various experimental groups.

### Cell cycle analysis

After cell transfection, a total of 10^6^ cells were harvested and suspended in 70% ethanol, then stored at 4°C overnight. After PBS rinsing, 100 μL of RNase A was added to the cell suspension and incubated in a water bath at 37°C for 30 minutes. Subsequently, 400 μL of propidium iodide (PI) was introduced into the mixture, which was then subjected to an additional incubation period at 4°C, shielded from light, lasting for 30 minutes. The ensuing cell suspension was subsequently subjected to flow cytometry analysis, thereby facilitating the assessment of the cellular distribution spanning various phases of the cell cycle.

### Tumor xenografts in nude mice

BALB/cA-nu mice, aged 4–6 weeks, were procured from HFK Bioscience (Beijing, China). Each mouse was subcutaneously injected with 106 transfected cells in the axillary region. Animal experiments were conducted in strict accordance with the protocol approved by the Scientific Research and New Technology Ethical Committee of the Shengjing Hospital of China Medical University. The graft volume was assessed using the following formula: tumor volumes (mm3) = length × width^2^/2. Tumor measurements were recorded every 4 days until 28 days later when the mice were euthanized. The tumors were fixed in a 4% poly sound solution, subjected to dehydration, paraffin embedding, and finally cut into paraffin sections for subsequent immunohistochemical analysis.

### Availability of data and materials

All data generated or analyzed during this study are included in this article.

## Supplementary Materials

Supplementary Tables
