# Supplementary material for: Analysis and experimental validation of fatty acid metabolism-related genes prostacyclin synthase (PTGIS) in endometrial cancer
Source: Aging (Albany NY). 2023 Oct 4;15(19):10322–46. doi: 10.18632/aging.205080 (PMC10599728; doi:10.18632/aging.205080)
Supplement: Supplementary Tables [file aging-15-205080-s001.pdf]

## SUPPLEMENTARY TABLES

**Supplementary Table 1. Primer sequence.**

| Gene name | Primer sequence |
|---|---|
| PTGIS | Forward: AAAGTCGCCTGTGGAAGCTG<br>Reverse: TGCCTGCATCTCCTCTGACA |
| GAPDH | Forward: CAGGAGGCATTGCTGATGAT<br>Reverse: GAAGGCTGGGGCTCATTT |

**Supplementary Table 2. Sequence of siRNA.**

| Name | Sequence |
|---|---|
| PTGIS-Homo-544 | Sense (5′–3′): GCCGGCUACCUGACUCUUUTT<br>Antisense (5′–3′): AAAGAGUCAGGUAGCCGGCTT |
